# Full-BAPose: Bottom Up Framework for Full Body Pose Estimation

**DOI:** 10.3390/s23073725

**Published:** 2023-04-04

**Authors:** Bruno Artacho, Andreas Savakis

**Affiliations:** Department of Computer Engineering, Rochester Institute of Technology, Rochester, NY 14623, USA

**Keywords:** human pose estimation, whole-body pose estimation, deep learning, multi-scale representations, waterfall module, atrous spatial pooling, adaptive convolutions, disentangled keypoint regression

## Abstract

We present Full-BAPose, a novel bottom-up approach for full body pose estimation that achieves state-of-the-art results without relying on external people detectors. The Full-BAPose method addresses the broader task of full body pose estimation including hands, feet, and facial landmarks. Our deep learning architecture is end-to-end trainable based on an encoder-decoder configuration with HRNet backbone and multi-scale representations using a disentangled waterfall atrous spatial pooling module. The disentangled waterfall module leverages the efficiency of progressive filtering, while maintaining multi-scale fields-of-view comparable to spatial pyramid configurations. Additionally, it combines multi-scale features obtained from the waterfall flow with the person-detection capability of the disentangled adaptive regression and incorporates adaptive convolutions to infer keypoints more precisely in crowded scenes. Full-BAPose achieves state-of-the art performance on the challenging CrowdPose and COCO-WholeBody datasets, with AP of 72.2% and 68.4%, respectively, based on 133 keypoints. Our results demonstrate that Full-BAPose is efficient and robust when operating under a variety conditions, including multiple people, changes in scale, and occlusions.

## 1. Introduction

Full body pose estimation is a challenging task in computer vision that finds applications in action recognition, human–computer interaction, and sign language recognition. Human pose estimation methods have received considerable attention for 2D pose estimation, including multistage architectures, such as stacked hourglass (HG) networks [1] and Convolutional Pose Machines (CPMs) [2], and encoder-decoder architectures, such as UniPose [3] and High-Resolution Network (HRNet) [4]. Multi-person pose estimation is particularly challenging due to joint occlusions and the large number of degrees of freedom in the human body. State-of-the-art (SOTA) methods for multi-person pose estimation can be top-down or bottom-up. Top-down approaches use detectors to localize instances of persons in the image and then perform single-person pose estimation for each instance. While top-down approaches generally achieve higher accuracy, they require external detectors that make the process slower and more costly. Bottom up methods either detect all keypoints and group them for each individual [5] or directly regress the keypoints to each person in the image [6]. Thus, bottom-up approaches require a single processing stage and are more efficient.

**Figure 1:**
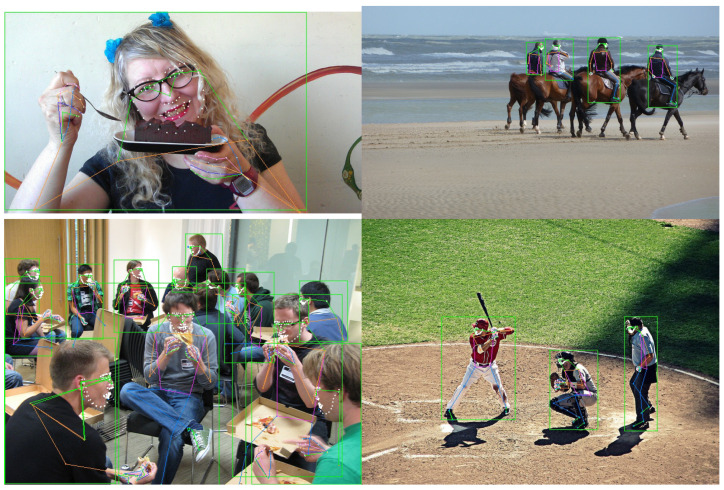
Full-body pose estimation examples with our Full-BAPose method showing a single person and multiple people at various scales and occlusions. The bottom-up approach determines the bounding boxes (in green) and the person pose estimation.

Full-BAPose is a full-body version of BAPose [7]. It is a bottom-up framework named after “Basso verso l’Alto” (bottom-up in Italian). The Full-BAPose method deals with the broader task of pose estimation for the whole body and is based on 133 keypoints for the body, hands, feet, and facial landmarks. Examples of Full-BAPose pose estimation are shown in Figure 1, illustrating the method’s effectiveness under various conditions. The Full-BAPose network is single-stage and end-to-end trainable, building upon the successful approaches by UniPose for single-person 2D pose [3], UniPose+ for single-person 2D and 3D pose [8], and OmniPose for multi-person top-down pose [9]. Full-BAPose achieves SOTA results without requiring post-processing, intermediate supervision, multiple iterations, or anchor poses. The main contributions of Full-BAPose are:

- A single-pass, end-to-end trainable, multi-scale approach for a bottom-up multi-person full-body pose estimation framework that achieves SOTA results;
- Our bottom-up approach operates without requiring a separate detector because it combines multi-scale waterfall features with disentangled adaptive convolutions and includes a decoder to determine the joints of individuals in crowded scenes.

## 2. Related Work

Convolutional Neural Networks (CNNs) have enabled impressive advances for human pose estimation [5,8,10,11]. Convolutional pose machines [2] utilize a CNN with stages that refine joint detection. Part Affinity Fields (PAFs) in OpenPose [5] capture relationships between joints for improved human pose estimation. The stacked hourglass network [1] cascades hourglass structures for pose estimation refinement. The multi-context approach in [12] augments the backbone with residual units to increase the receptive Field-of-View (FOV) but increases complexity due to postprocessing with Conditional Random Fields (CRFs). Aiming to develop a unified framework for single-person pose estimation, UniPose [3] and UniPose+ [8] combine bounding box generation and joint detection in a unified, one-pass network. This is made feasible by the Waterfall Atrous Spatial Pooling (WASP) module [13], which allows a larger field-of-view and multi-scale representations to determine context.

The multi-scale approach of HRNet includes both high and low resolutions to obtain a larger FOV, while higher HRNet [14] combined the HRNet structure with multi-resolution pyramids. The Multi-Stage Pose Network (MSPN) [15] combines the cross-stage feature aggregation and coarse-to-fine supervision. The Distribution-Aware coordinate Representation of Keypoints (DARK) method [16] refines their decoder in order to reduce the inference error at the decoder stage.

The work in [17] utilized graphs to extract contextual information for pose. Cascade Feature Aggregation (CFA) [18] employed the cascade approach for semantic information in pose estimation. Generative Adversarial Networks (GANs) were used in [19] to learn context for poses. More recently, methods such as TokenPose [20] are investigating transformer networks to determine global dependencies for pose estimation. Neural architecture search was explored in ZoomNAS [21] to obtain separate networks for pose estimation on the body, hands, and face.

A limitation of top-down approaches is the requirement of an independent module for the detection of instances of humans in the frame. LightTrack [22], for instance, applies YOLOv3 [23] to detect subjects prior to the detection of joints for pose estimation, while LCR-Net [10] applies multiple branches for detection by using the Detectron [24] and the arrangement of joints during classification.

### 2.1. Bottom-Up Approaches

Bottom-up approaches face the bigger challenge of detecting the joints of multiple people without an external person detector used by top-down methods. Bottom-up methods try to associate detected keypoints with persons in the image. This problem was cast in [25,26] as integer linear programming, but this type of optimization requires a significant processing time. OpenPose [5] used part affinity fields in a breakthrough approach to grouping keypoints for each person. This approach was extended by Pif-Paf [27] and associative embedding [28]. PersonLab [29] adopted Hough voting and [30] Hierarchical Graphical Clustering (HGG). The works in [31,32] consider dense regression of pose candidates but face the limitation of lower localization accuracy that requires post-processing to improve the results. In a related approach, Ref. [33] considered a mixture density network before regression. Alternatively, the Disentangled Keypoint Regression (DEKR) [6] method learns disentangled representations for each keypoint and utilizes adaptively activated pixels so that each representation focuses on the corresponding keypoint area.

### 2.2. Multi-Scale Feature Representations

The pooling operations in CNNs present a challenge for pose estimation due to the resolution reduction. To overcome this problem, Fully Convolutional Networks (FCNs) [34] utilize upsampling to increase the resolution of the feature maps to the size of the input image. DeepLab [35] adopted atrous convolutions in the multi-scale Atrous Spatial Pyramid Pooling (ASPP) framework that maintains the size of the receptive fields. DeepLab applies atrous convolutions in four parallel branches with different rates and combines them at the original image resolution.

Presenting an improvement over ASPP, the WASP module [3,13] generates multi-scale features in an efficient manner by creating a waterfall flow. Instead of immediately parallelizing all branches of the input stream, the WASP module first processes through a filter and then creates a new branch. The waterfall flow extends the cascade approach by combining the streams from all its branches to create a multi-scale representation.

## 3. Full-BAPose Architecture

**Figure 2:**
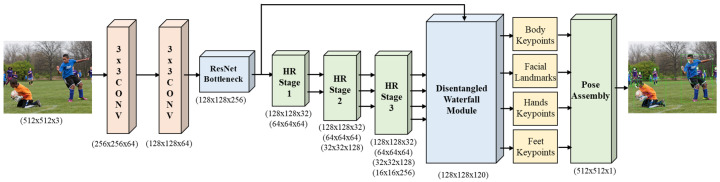
Full-BAPose architecture for whole-body multi-person pose estimation. The input color image is fed through the HRNet backbone for initial feature extraction. The feature sizes are denoted by the two spatial dimensions first and the channel dimension last, e.g., (128 × 128 × 32) denotes feature size of 128 × 128 with 32 channels. The HRNet features are combined by the D-WASP module and a decoder utilizing adaptive convolutions generates the detection bounding boxes and the keypoints for the hand, head, feet, and entire body, i.e., 133 keypoints and 4 bounding boxes for each person instance.

The Full-BAPose bottom-up architecture, as illustrated in Figure 2, consists of a single-pass, end-to-end trainable network with HRNet backbone and our Disentangled Waterfall Atrous Spatial Pyramid (D-WASP) module with an integrated decoder for full-body multi-person pose estimation. The input image is fed in the HRNet backbone and the extracted feature maps from the HRNet levels are processed by the D-WASP module to obtain enhanced multi-scale representations. The D-WASP module includes an integrated decoder to obtain the location of keypoints and contextual information for the localization regression. The network generates *K* heatmaps, one for each joint, with the corresponding confidence maps, as well as two offset maps for the identification of person instances and the association of keypoints to each instance. The integrated D-WASP decoder generates detections from all scales of the feature extraction for both visible and occluded joints while maintaining the image resolution throughout the network.

Our approach includes several innovations that contribute to increased accuracy. The D-WASP module combines atrous convolutions and the waterfall architecture to improve multi-scale representations with contextual information by the processing of feature maps at multiple rates of dilation. Our architecture allows a larger FOV in the encoder and integrates disentangled adaptive convolutions in the decoder, facilitating the detection of multi-person instances and their keypoints in a single-pass. Further, our network demonstrates the ability to deal with a large number of persons in the image due to feature extraction at multiple scales. Finally, the modular nature of the architecture facilitates easy implementation and training of the network.

Full-BAPose utilizes a bottom-up approach without relying on external detectors to locate faces or people. The D-WASP module combines the multi-scale approach of the waterfall atrous convolutions with disentangled adaptive convolutions to better estimate the joints and effectively detect multiple person instances.

### 3.1. Disentangled Waterfall Module

**Figure 3:**
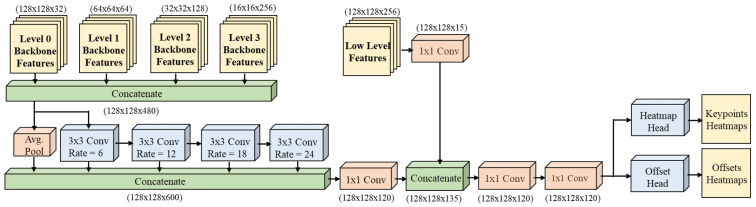
The D-WASP disentangled waterfall module. The feature sizes are denoted by the two spatial dimensions, followed by the channel dimension. The inputs are 32, 64, 128, and 256 feature maps from all four levels of the HRNet backbone, as illustrated in Figure 2, and low-level features from the initial layers of the framework. The module processes the backbone features at different rates of dilation in a waterfall fashion and outputs both the keypoints and offset heatmaps for each person instance.

The enhanced “Disentangled Waterfall Atrous Spatial Pyramid” module is shown in Figure 3. The D-WASP module processes all four levels of feature maps from the backbone through the waterfall branches with different dilation rates for multi-scale representations. Low-level and high-level features are represented at the same resolution, achieving refined joint estimation. Furthermore, the D-WASP module uses adaptive convolution blocks to infer heatmaps for joint localization and offset maps for person instance regression.

The design of the D-WASP module relies on a combination of atrous and adaptive convolutions. Atrous convolutions are utilized in the initial stages to expand the FOV by performing a filtering cascade at increasing rates to gain efficiency. The waterfall modules are designed to create a waterfall flow, initially processing the input and then creating a new branch. D-WASP goes beyond the cascade approach of [36] by combining all streams from all its branches and the average pooling layer from the original input. Additionally, our module incorporates a larger number of scales compared to previous versions of the waterfall module [3,9] by adopting all 480 feature maps from all levels of the HRNet feature extractor. Adaptive convolutions are used for improved estimation of individual keypoints and offset heatmaps during the regression process by providing context around each detected joint and associated joints.

#### 3.1.1. Waterfall Features and Adaptive Convolutions

The D-WASP module operation starts with the concatenation $g_{0}$ of all feature maps $f_{i}$ from the HRNet feature extractor, where i=0,1,2,3 indicates the levels at different scales and summation indicates concatenation:

(1) $$g_{0}=\sum_{i=0}^{3}\left(f_{i}\right)$$

The waterfall processing is described as follows:

(2) $$f_{Waterfall}=W_{1}⊛\left(\sum_{i=1}^{4}\left(W_{d_{i}}⊛g_{i-1}\right)+AP\left(g_{0}\right)\right)$$

(3) $$f_{maps}=W_{1}⊛(W_{1}⊛\left(W_{1}⊛f_{LLF}+f_{Waterfall}\right)$$

where ⊛ represents convolution, $g_{0}$ is the input feature map, $g_{i}$ is the feature map from the *i*th atrous convolution, AP is the average pooling operation, $f_{LLF}$ are the low-level feature maps, and $W_{1}$ and $W_{d_{i}}$ represent convolutions of kernel size 1 × 1 and 3 × 3 with dilations of $d_{i}=\left[1,6,12,18\right]$, as shown in Figure 3. After concatenation, the feature maps are combined with low-level features. The last 1 × 1 convolution reduces the number of feature maps down to one quarter of the number in the combined input feature maps.

Finally, the D-WASP module output $f_{D-WASP}$ is obtained from the multi-scale adaptive convolutional regression, where adaptive convolution is defined as:

(4) $$y\left(c\right)=\sum_{i=1}^{9}\left(w_{i}x\left(g_{i}^{c}+c\right)\right)$$

where *c* is the center pixel of the convolution, y(c) represents the output of the convolution for input *x*, $w_{i}$ are the kernel weights for the center pixel and its neighbors, and $g_{i}^{c}$ is the offset of the *i*th activated pixel. In the adaptive convolutions, the offsets $g_{i}^{c}$ are adopted in a parametric manner as an extension of spatial transformer networks [37].

#### 3.1.2. Disentangled Adaptive Regression

The regression stage for multi-person pose estimation is a bottleneck in performance for bottom-up methods. To address this limitation, additional processing may utilize pose candidates, post-processing matching, proximity matching, or statistical methods. However, these may be computationally expensive or limited in effectiveness.

D-WASP leverages the idea of regression by focus by learning disentangled representations for each of the *K* joints and using multiple scales to infer each representation for all keypoints from multiple adaptively activated pixels. This configuration offers more robust contextual information of the keypoint region and results in a more accurate spatial representation. In addition, the integration of the multi-scale feature maps in the disentangled adaptive regression utilizes multiple resolutions at the regression stage, allowing the network to better infer the locations of people and their joints.

## 4. Datasets

We evaluated BAPose on two datasets for 2D multi-person pose estimation: Common Objects in Context (COCO) [38] and CrowdPose [39]. The large and most commonly adopted COCO dataset [38] consists of over 200K images with more than 250K instances of labelled people keypoints. The keypoint labels consist of 17 keypoints including all major joints in the torso and limbs, as well as facial landmarks, including nose, eyes, and ears. The dataset is challenging due to the large number of images in a diverse set of scales and occlusion for poses in the wild.

The CrowdPose dataset [39] is more challenging due to crowds and low separation among individuals. The dataset contains 10K images for training, 2K images for validation, and 20K images for testing. In addition to joint annotations, it also contains body part occlusions. We follow evaluation procedures from [6,14].

The COCO-WholeBody dataset consists of images from the large COCO dataset labeled to contain facial landmarks, feet and hands keypoints, and the original body keypoints, totaling 133 keypoints to be extracted by the network for multiple people in each image. The dataset contains over 130K instances of facial landmarks, hands, and feet for a total of over 800K hand keypoints and 1.4M facial landmarks.

We generated ideal Gaussian maps for the joints’ ground truth locations during training, which is a more effective strategy for training loss assessment compared to single points at joint locations. As a consequence, the BAPose output heatmap locations for each joint. The value of σ=3 was adopted, generating a well-defined Gaussian response for both the ground truth and keypoint predictions, with a decent separation of keypoints and avoidance of large overlapping of keypoints.

## 5. Experiments

BAPose experiments followed standard metrics set by each dataset and the same procedures applied by [6,14].

### 5.1. Metrics

For the evaluation of BAPose, the evaluation is conducted based on the Object Keypoint Similarity metric (OKS).

(5) $$\mathrm{OKS}=\frac{\left(\sum_{i}e^{-d_{i}^{2}/2s^{2}k_{i}^{2}}\right)\delta \left(v_{i}>0\right)}{\sum_{i}\delta \left(v_{i}>0\right)}$$

where $d_{i}$ is the Euclidian distance between the estimated keypoint and its ground truth, $v_{i}$ indicates if the keypoint is visible, *s* is the scale of the corresponding target, and $k_{i}$ is the falloff control constant. Since the OKS measurement is adopted by both datasets and is similar to the intersection over the union (IOU), we report our OKS results as the Average Precision (AP) for the IOUs for all instances between 0.5 and 0.95 (AP), at 0.5 ($AP^{50}$) and 0.75 ($AP^{75}$), as well as instances of medium ($AP^{M}$) and large size ($AP^{L}$) for the COCO dataset. For the CrowdPose dataset, we report easy ($AP^{E}$), medium ($AP^{M}$), and hard size ($AP^{H}$) instances, as well as the overall Average Recall (AR), including for medium ($AR^{M}$) and large ($AR^{L}$) instances.

### 5.2. Parameter Selection

We use a set of dilation rates of r= {1, 6, 12, 18} for the D-WASP module, similar to [9], and train the network for 140 epochs. The learning rate is initialized at $10^{-3}$ and is reduced by an order of magnitude in two steps at 90 and 120 epochs. The training procedure includes random rotation $\left[-30^{\circ },30^{\circ }\right]$, random scale [0.75,1.5], and random translation [−40,40], mirroring procedures followed by [6]. All experiments were performed using PyTorch on Ubuntu 16.04. The workstation has an Intel i5-2650 2.20 GHz CPU with 16 GB of RAM and an NVIDIA Tesla V100 GPU.

## 6. Results

This section presents body pose results with BAPose [7] and full-body pose results with Full-BAPose, as well as comparisons with SOTA methods.

### 6.1. Experimental Results on the CrowdPose Dataset

**Figure 4:**
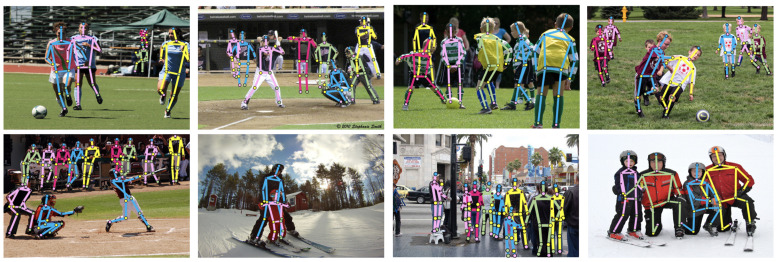
Pose estimation examples using BAPose with the CrowdPose dataset.

We performed training and testing of BAPose (for body pose based on 17 keypoints) on the CrowdPose dataset, a difficult challenge due to the high occurrence of crowds in the images. The results are shown in Table 1. Our BAPose-W32 method uses the HRNet-W32 backbone [6]. Figure 4 illustrates successful detections of multi-person pose for the CrowdPose test set. The examples demonstrate how effectively BAPose deals with occlusions, the close proximity of individuals, as well as detections at different scales.

BAPose significantly improves upon SOTA methods for 512 × 512 input resolution, achieving an AP of 72.2%. BAPose outperforms other bottom-up approaches by a wide margin, even those that utilized higher input resolutions. BAPose increased the AP of previous bottom-up methods from 65.7% to 72.2% (relative increase of 9.9%) when compared to previous SOTA at the same resolution, which is a 19.0% reduction in error (from 34.3% to 27.8%). The capabilities of the multi-scale approach of BAPose are further illustrated by observing more precise joint estimations with a threshold of 75% ($AP^{75}$), drastically reducing the error by 25.7% (from 29.6% to 22.0%) and increasing the previous SOTA AP from 70.4% to 78.0% (relative increase of 10.8%) when compared to the previous SOTA HRNet-W32 [6].

Additionally, BAPose outperforms networks that utilize top-down approaches by a significant margin, increasing from 70.0% to 72.2%. In contrast to top-down methods, BAPose does not rely on ground truth for person detection and has to infer the location of all individuals in a modular, single-pass process. For the CrowdPose dataset, BAPose’s performance is superior to networks utilizing higher-resolution inputs of 640 × 640 [6,14] while processing the less computationally expensive 512 × 512 resolution.

We observe that the BAPose framework was able to achieve this significant increase in AP for the CrowdPose dataset while utilizing a backbone that is smaller (HRNet-W32) compared to the previous SOTA deploying a larger backbone (HRNet-W48 [6]), reducing the number of parameters by 54.9% and GFLOPs by 67.9%.

### 6.2. Experimental Results on the COCO Dataset

We next performed training and testing of BAPose on the COCO dataset, which is challenging due to the large number of diverse images with multiple people in close proximity and images lacking a person instance. We first compared BAPose with SOTA methods for the COCO validation and test-dev datasets. The validation results in Table 2 show that BAPose achieves significant improvement over the previous SOTA for both input resolutions. Our BAPose-W32 and BAPose-W48 methods use HRNet-W32 and HRNet-W48 backbones respectively [6]. The BAPose results at the former resolution are obtained with a significantly lower computational cost compared to methods with a higher resolution, as shown in Table 3, while achieving comparable results to a higher resolution.

The incorporation of the D-WASP module achieves an increased overall accuracy of 69.1% when using single-scale testing, significantly increasing the AP accuracy at 512 × 512 resolution by 1.6%. For multi-scale testing, BAPose achieves 71.9%, improving upon previous SOTA of 70.7%, which is an increase in accuracy of 1.7%. This performance increase represents an error reduction of 3.4% (from 32.0% to 30.9%) for single scale and 4.1% error reduction for multi-scale (from 29.3% to 28.1%).

BAPose improves the accuracy of the previous SOTA in all keypoint estimation metrics and IOU for the COCO dataset. Most of the performance improvements of BAPose are attributed to performing better on harder detections and more refined predictions at $AP^{75}$. The results on the COCO validation dataset, in Table 2, show the greater capability of BAPose to detect more complex and harder poses while still using a smaller resolution in the input image.

We also trained and tested BAPose-W48 at a 640 × 640 resolution, achieving 71.6% accuracy for the COCO validation set with single-scale testing and 72.7% with multi-scale testing, improving the previous SOTA by 0.8% and 0.6%, respectively. This improvement represents an error reduction of 2.1% and 1.4% compared to HRNet-w48 [6]. However, larger resolution models require much higher computational resources, as illustrated by the GFLOPs and memory requirements in Table 3. Compared to BAPose-W32, HRNet-W48 requires a 249.1% increase in the number of GFLOPs, and HigherHRNet-W48 requires a 271.7% increase in the number of GFLOPs, demonstrating that BAPose-W32 results in a better trade-off between accuracy and computational cost.

**Figure 5:**
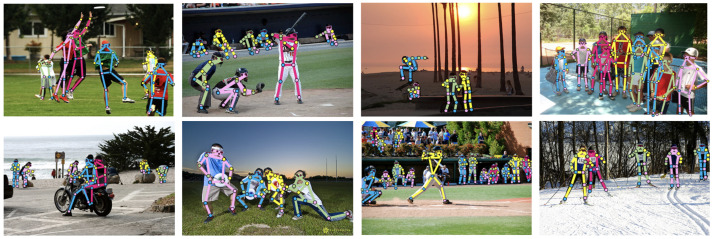
Pose estimation results using BAPose with the COCO dataset.

Figure 5 presents examples of pose estimation results for the COCO dataset. BAPose effectively locates symmetric body joints and avoids confusion due to occlusion between individuals. This is illustrated in harder-to-detect joints, such as ankles and wrists. Overall, the BAPose results demonstrate robustness for pose estimation in challenging conditions, such as images that include multiple individuals with a high overlapping ratio combined with shadows or darker images or partial poses present in the image.

For the larger COCO test-dev dataset shown in Table 4, BAPose achieves again new SOTA performance over methods using input resolutions of 512 × 512. Our method obtained an overall precision of 68.0% when using single-scale testing and 70.4% when using multi-scale testing, which are relative improvements over SOTA of 1.0% for single (from 67.3% to 68.0%) and 1.1% (from 69.6.% to 70.4%) for multi scale testing. BAPose reduced the error at the 512 × 512 resolution by 2.1% (from 32.7% to 32.0%) for single-scale and 2.6% (from 30.4% to 29.6%) for multi-scale testing. When training and testing at the 640 × 640 resolution, BAPose-W48 achieved accuracies of 70.3% for single-scale testing and 71.2% when using single-scale multi-scale testing, an improvement of 0.4% for single-scale testing and 0.3% for multi-scale testing compared to the previous SOTA, reducing the error by 1.0% and 0.7%, respectively. These results further demonstrate BAPose’s most significant improvements are in smaller and harder targets consistent with the findings from the validation dataset.

### 6.3. Experimental Results on the COCO-WholeBody Dataset

We trained and tested Full-BAPose on the COCO-WholeBody dataset [44] for the larger task of estimating a full set of keypoints including all body pose keypoints, all joints of feet and hands, and facial landmarks.

The comparison of both the Full-BAPose and OmniPose framework to state-of-the-art methods for the validation dataset is shown in Table 5. The results demonstrate that both architectures present a significant increase compared to the previous state-of-the-art, especially the Full-BAPose framework, achieving an accuracy increase of 13.1% in the overall accuracy compared to the previous state-of-the-art. In addition, it is important to notice that the large increase observed by Full-BAPose utilizes a shared and unique backbone to detect all 133 keypoints in contrast to previous work that deploy different backbones for different tasks (face, body, hands, feet) on the COCO-WholeBody dataset.

**Figure 6:**
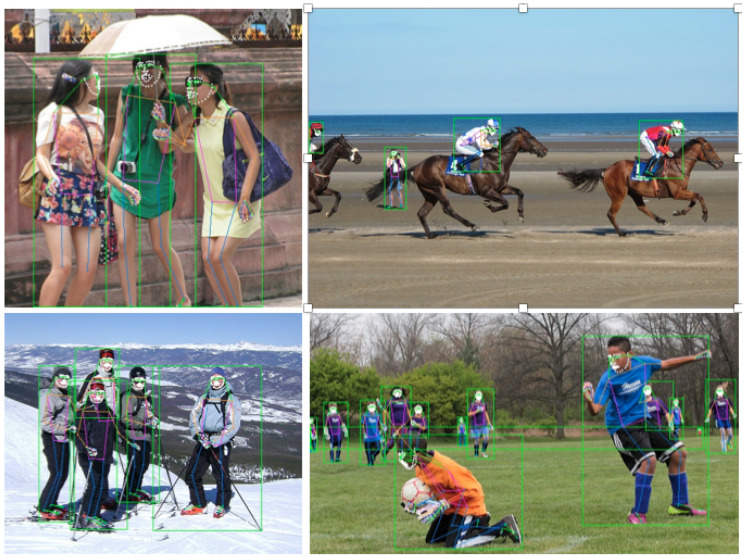
Pose estimation results using Full-BAPose with the COCO-WholeBody dataset.

Figure 6 presents sample pose estimation for the COCO-WholeBody dataset, exemplifying the high accuracy of Full-BAPose for the complete human pose estimation task.

## 7. Conclusions

We presented the Full-BAPose framework for bottom-up multi-person, full-body pose estimation. The Full-BAPose method addressed the broader task of full-body pose estimation including hands, feet, and facial landmarks. The Full-BAPose network includes the D-WASP module that combines multi-scale features obtained from the waterfall flow with the person-detection capability of disentangled adaptive regression. The results demonstrate SOTA performance on body pose for both the COCO and CrowdPose datasets in all metrics, as well as superior capability of person detection and pose estimation in densely populated images. Future work will extend our framework with vision transformers for improved performance.

## Figures and Tables

**Table 1:** BAPose results and comparison with SOTA methods for the CrowdPose dataset for testing. TP and BU represent the top-down and bottom-up approaches, respectively. Best results are in bold.

Method	Input Size	Approach	AP	${\mathrm{AP}}^{50}$	${\mathrm{AP}}^{75}$	${\mathrm{AP}}^{E}$	${\mathrm{AP}}^{M}$	${\mathrm{AP}}^{H}$
BAPose-W32 (ours)	512	BU	**72.2%**	**89.6%**	**78.0%**	**79.9%**	**73.4%**	**61.3%**
MIPNet [40]	512	TP	70.0%	-	-	-	-	-
HRNet-W48 [6]	640	BU	67.3%	86.4%	72.2%	74.6%	68.1%	58.7%
JC SPPE [39]	-	TP	66.0%	84.2	71.5	75.5%	66.3%	57.4%
HigherHRNet-W48 [14]	640	BU	65.9%	86.4%	70.6%	73.3%	66.5%	57.9%
HRNet-W32 [6]	512	BU	65.7%	85.7%	70.4%	73.0%	66.4%	57.5%
Mask R-CNN [41]	-	BU	60.3%	-	-	69.4%	57.9%	45.8%

**Table 2:** BAPose results and comparison with SOTA methods for the COCO dataset for validation. Best results are in bold.

Method	Input Size	AP	${\mathrm{AP}}^{50}$	${\mathrm{AP}}^{75}$	${\mathrm{AP}}^{M}$	${\mathrm{AP}}^{L}$	AR
Single-Scale Testing							
BAPose-W48 (ours)	640	**71.6%**	**88.6%**	**78.3%**	**67.3%**	**78.7%**	**76.5%**
HRNet-W48 [6]	640	71.0%	88.3%	77.4%	66.7%	78.5%	76.0%
HigherHRNet-W48 [14]	640	69.9%	87.2%	76.1%	-	-	-
BAPose-W32 (ours)	512	**69.1%**	**87.0%**	**75.6%**	**63.1%**	**78.6%**	**73.7%**
HRNet-W32 [6]	512	68.0%	86.7%	74.5%	62.1%	77.7%	73.0%
HigherHRNet-W32 [14]	512	67.1%	86.2%	73.0%	-	-	-
HGG [30]	512	60.4%	83.0%	66.2%	-	-	64.8%
Multi-Scale Testing							
BAPose-W48 (ours)	640	**72.7%**	**88.6%**	**79.1%**	**69.3%**	78.4%	**77.9%**
HRNet-W48 [6]	640	72.3%	88.3%	78.6%	68.6%	**78.6%**	77.7%
HigherHRNet-W48 [14]	640	72.1%	88.4%	78.2%	-	-	-
BAPose-W32 (ours)	512	**71.9%**	**88.3%**	**77.8%**	**67.2%**	**79.1%**	**76.6%**
HRNet-W32 [6]	512	70.7%	87.7%	77.1%	66.2%	77.8%	75.9%
HigherHRNet-W32 [14]	512	69.9%	87.1%	76.0%	-	-	-
HGG [30]	512	68.3%	86.7%	75.8%	-	-	72.0%

**Table 3:** GFLOPs and number of parameters comparison.

Method	Input Size	GFLOPs	Params (M)
HRNet-W32 [6]	512	45.4	29.6
BAPose-W32 (ours)	512	56.8	30.3
HRNet-W48 [6]	640	141.5	65.7
HigherHRNet-W48 [14]	640	154.3	63.8
BAPose-W48 (ours)	640	183.2	67.4

**Table 4:** BAPose results and comparison with SOTA methods for the COCO dataset for test-dev. Best results are in bold.

Method	Input Size	AP	${\mathrm{AP}}^{50}$	${\mathrm{AP}}^{75}$	${\mathrm{AP}}^{M}$	${\mathrm{AP}}^{L}$	AR
Single-Scale Testing							
BAPose-W48 (ours)	640	**70.3%**	**89.6%**	**77.5%**	**65.9%**	**77.1%**	**75.4%**
HRNet-W48 [6]	640	70.0%	89.4%	77.3%	65.7%	76.9%	75.4%
HigherHRNet-W48 [14]	640	68.4%	88.2%	75.1%	64.4	74.2	-
BAPose-W32 (ours)	512	**68.0%**	**88.0%**	**74.8%**	**62.4%**	**76.6%**	**72.9%**
HRNet-W32 [6]	512	67.3%	87.9%	74.1%	61.5%	76.1%	72.4%
SPM [31]	-	66.9%	88.5%	72.9%	62.6%	73.1%	-
CenterNet-HG [42]	512	63.0%	86.8%	69.6%	58.9%	70.4%	-
OpenPose [5]	-	61.8%	84.9%	67.5%	57.1%	68.2%	66.5%
Multi-Scale Testing							
BAPose-W48 (ours)	640	**71.2%**	**89.4%**	**78.1%**	**67.4%**	76.8%	**76.8%**
HRNet-W48 [6]	640	71.0%	89.2%	78.0%	67.1%	**76.9%**	76.7%
HigherHRNet-W48 [14]	640	70.5%	89.3%	77.2%	66.6%	75.8%	-
Point-set Anchors [43]	640	68.7%	89.9%	76.3%	64.8%	75.3%	74.8%
BAPose-W32 (ours)	512	**70.4%**	**89.3%**	**77.4%**	**66.0%**	**76.9%**	**75.6%**
HRNet-W32 [6]	512	69.6%	89.0%	76.6%	65.2%	76.5%	75.1%
HGG [30]	512	67.6%	85.1%	73.7%	62.7%	74.6%	71.3%

**Table 5:** Full-BAPose results and comparison with SOTA methods for the COCO-WholeBody dataset for validation. Best results are in bold.

Method	Backbone	Approach	Single	Whole Body	Body	Foot	Face	Hand
			Task	AP	AP	AP	AP	AP
Full-BAPose	HRNet-W48	Top-Down	N	**68.4%**	**74.4%**	76.4%	**86.8%**	**64.6%**
OmniPose [9]	HRNet-W48	Top-Down	N	65.8%	73.8%	66.6%	86.8%	60.2%
Zauss et al. [45]	ShuffleNetV2k168	Bottom-Up	N	60.4%	69.6%	63.4%	85.0%	52.9%
ZoomNet [44]	2 × HRNet (W32 + W18)	Top-Down	Y	54.1%	74.3%	**79.8%**	62.3%	40.1%
HRNet [4]	HRNet-W32	Top-Down	N	43.2%	65.9%	31.4%	52.3%	30.0%
HPRNet [46]	HG	Bottom-Up	N	34.8%	59.4%	53.0%	75.4%	50.4%
OpenPose [5]	-	Bottom-Up	N	33.8%	56.3%	53.2%	48.2%	19.8%
AE [28]	HG	Bottom-Up	N	27.4%	40.5%	7.7%	47.7%	34.1%

## Abbreviations

The following abbreviations are used in this manuscript:

AP	Average Precision
AR	Average Recall
ASPP	Atrous Spacial Pyramid Pooling
CFA	Cascade Feature Aggregation
CNN	Convolutional Neural Networks
COCO	Common Objects in Context
CPM	Convolutional Pose Machines
CRF	Conditional Random Fields
DARK	Distribution-Aware coordinate Representation of Keypoints
DEKR	Disentangled Keypoint Regression
FCN	Fully Convolutional Networks
FOV	Field-of-View
GAN	Generative Adversarial Networks
HG	Hourglass
HGG	Hierarchical Graphical Clustering
HRNet	High-Resolution Network
IOU	Intersection Over the Union
MSPN	Multi-Stage Pose Network
OKS	Object Keypoint Similarity
PAF	Part Affinity Fields
SOTA	State-of-the-art
WASP	Waterfall Atrous Spatial Pooling
